# Aconitase B Is Required for Optimal Growth of *Xanthomonas campestris* pv. *vesicatoria* in Pepper Plants

**DOI:** 10.1371/journal.pone.0034941

**Published:** 2012-04-06

**Authors:** Janine Kirchberg, Daniela Büttner, Barbara Thiemer, R. Gary Sawers

**Affiliations:** 1 Department of Microbiology, Institute of Biology, Martin-Luther University Halle-Wittenberg, Halle (Saale), Germany; 2 Department of Genetics, Institute of Biology, Martin-Luther University Halle-Wittenberg, Halle (Saale), Germany; Friedrich-Alexander-University Erlangen-Nurenberg, Germany

## Abstract

The aerobic plant pathogenic bacterium *Xanthomonas campestris* pv. *vesicatoria* (*Xcv*) colonizes the intercellular spaces of pepper and tomato. One enzyme that might contribute to the successful proliferation of *Xcv* in the host is the iron-sulfur protein aconitase, which catalyzes the conversion of citrate to isocitrate in the tricarboxylic acid (TCA) cycle and might also sense reactive oxygen species (ROS) and changes in cellular iron levels. *Xcv* contains three putative aconitases, two of which, *acnA* and *acnB*, are encoded by a single chromosomal locus. The focus of this study is aconitase B (AcnB). *acnB* is co-transcribed with two genes, XCV1925 and XCV1926, encoding putative nucleic acid-binding proteins. *In vitro* growth of *acnB* mutants was like wild type, whereas *in planta* growth and symptom formation in pepper plants were impaired. While *acnA,* XCV1925 or XCV1926 mutants showed a wild-type phenotype with respect to bacterial growth and *in planta* symptom formation, proliferation of the *acnB* mutant in susceptible pepper plants was significantly impaired. Furthermore, the deletion of *acnB* led to reduced HR induction in resistant pepper plants and an increased susceptibility to the superoxide-generating compound menadione. As AcnB complemented the growth deficiency of an *Escherichia coli* aconitase mutant, it is likely to be an active aconitase. We therefore propose that optimal growth and survival of *Xcv* in pepper plants depends on AcnB, which might be required for the utilization of citrate as carbon source and could also help protect the bacterium against oxidative stress.

## Introduction

Pathogenic bacteria of the genus *Xanthomonas* infect both mono- and dicotyledonous plants and they are responsible worldwide for considerable losses in plant productivity [1], [2], [3]. *Xanthomonas campestris* pv. *vesicatoria* (*Xcv*) causes bacterial spot disease on pepper and tomato plants and is a model bacterium for the study of bacterial pathogenesis [1]. It enters the plant through openings such as wounds or through stomata and colonizes the intercellular spaces between plant cells. Virulence of the bacterium depends on the type III secretion system (T3SS) [4], [5], which injects a number of effector proteins into plant cells. As well as being involved in the development of disease symptoms, many of these effector proteins probably interfere with the host defense mechanisms. To establish effective colonization of the host the bacterium not only has to acquire growth substrates successfully but presumably also has to deal with a number of defense responses initiated by the host in response to infection. Amongst these, iron-restriction and an induced oxidative stress response are likely to be important [6], [7].

Comparatively little is known regarding how *Xcv* grows *in planta*, particularly with regard to substrate utilization but also with regard to the strategies employed to combat host defense mechanisms. Because *Xcv* is an obligate aerobe, this significantly increases the spectrum of carbon sources available to the bacterium for biosynthesis of new cell material and energy conservation. These carbon sources include a number of organic acids and amino acids, which can be oxidized by the tricarboxylic acid (TCA) cycle. Current evidence indicates that citrate is one important organic acid that can be used by the bacterium as a carbon source in the plant apoplast [8], [9], [10]. A key enzyme of the TCA cycle that not only catalyzes the interconversion of citrate and isocitrate, but also has a role in monitoring iron homeostasis and sensing oxidative stress is aconitase (Acn). Members of the Acn protein family are large monomeric, or occasionally dimeric [11], proteins that have a labile [4Fe-4S] cluster, which is required for enzyme activity. Because of the labile nature of the [4Fe-4S] cluster aconitases can function as sensors of both iron limitation and oxidative stress and this has meanwhile been demonstrated for a number of organisms [12]. Upon disassembly of the [4Fe-4S] cluster the apo-protein (termed the iron-responsive protein, IRP) adopts an alternative conformation that allows it to regulate gene expression at a post-transcriptional level. Processes regulated by apo-Acn include the oxidative stress response [13], sporulation in *Bacillus subtilis* [14] and stationary phase survival in *Staphylococcus aureus* [15]. A close association between Acn, iron deficiency and bacterial virulence has also been demonstrated for several bacterial pathogens [16], [17].

Bacterial Acns fall into two main classes, AcnA and AcnB [12], [18]. Although AcnA and AcnB have related biochemical activities [19], they exhibit only limited amino acid sequence similarity with each other. They have different domain organisation and AcnB proteins have an extra dimerization domain required both for protein-protein interaction and mRNA-binding activity [20]. In many bacteria AcnB is the main aconitase functional in the TCA cycle and it is sensitive to oxidative stress. AcnA on the other hand is induced in the stationary phase in response to iron and oxidative stresses [21], [22].

Circumstantial evidence for an important role of aconitase in regulating pathogenicity factor gene expression, e.g. production of extracellular enzymes and polysaccharides, in the plant pathogenic bacterium *X. campestris* pv. *campestris* was provided when an analysis of an *rpfA* mutant (regulation of pathogenicity factors A) proved to have a mutation in the gene encoding AcnA [23]. Comparative genome analysis reveals that both *X. campestris* pv. *campestris* and *Xcv* each encode three Acns [24], [25]. An *acnA* gene, equivalent to *rpfA* [23], is divergently transcribed from the *acnB* gene while a second *acnA2* gene (XCV1158) is located at a separate location on the genome in a cluster of genes predicted to encode enzymes of methylcitrate metabolism [24]. In this study we examined the potential role of AcnB in the pathogenesis and growth of *Xcv in planta*. Our studies reveal a requirement for AcnB to allow optimal growth of *Xcv* in pepper plants but not in liquid culture.

## Results

### The XCV1925-XCV1926*-acnB* Genes from *Xcv* are Co-transcribed and Conserved in the Genus *Xanthomonas*

**Figure 1:**
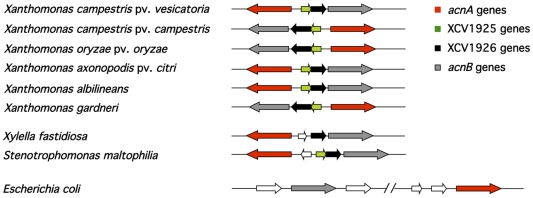
Schematic overview of the *acnA-xcv1925-xcv1926-acnB* locus in different *Xanthomonas* species. Shown is the organisation of the *acnA* and *acnB* genes, together with XCV1925 and XCV1926 which encode proteins of unknown function. The colour key for the genes is shown in the upper right of the figure. The synteny of the *acnAB* locus of *Xcv* was used as a comparator with other members of the *Xanthomonadales*. The genes represented by white arrows indicate genes with products unrelated to aconitases or XCV1925 or XCV1926 and the parallel, sloping lines in the *E. coli* genome representation denote physical separation of the two loci. Note that the genes are not drawn to scale. The accession numbers of the strains are as follows: *Xcv* strain 85-10, AM039952; *X. campestris pv. campestris* str. 8004, CP000050; *X. oryzae* pv. *oryzae* str. KACC10331, AE013598; *X. axonopodis* pv. *citri* str. 306, AE008923; *X. albilineans* str. GPE PC73, FP565176; *X. gardneri* ATCC 19865, AEQX00000000 annotation incomplete [61]; *Xylella fastidiosa* str. M-23, CP000941; *Stenotrophomonas maltophilia* str. K279a, AM743169; *E. coli*, CP001509.

The genes encoding AcnA and AcnB are divergently transcribed in the genus *Xanthomonas* (Fig. 1). Immediately upstream of *acnB* are two genes, termed XCV1925 and XCV1926. While XCV1926 is conserved in all species of the genus *Xanthomonas*, XCV1925 is absent in *Xanthomonas fuscans*. A similar gene organisation is also observed in the plant pathogenic bacterium *Xylella fastidiosa* and in *Stenotrophomonas maltophilia*, both of which belong to the family *Xanthomonadaceae*. This gene order is not conserved in other gammaproteobacteria such as *Escherichia coli* (Fig. 1).

The XCV1925 gene encodes a predicted protein of 8.7 kDa that belongs to the AbrB family of transition-state regulators [26], while XCV1926 encodes a predicted member of the VapC/PIN family of ribonucleases [27], [28]. AbrB proteins respond to a variety of environmental stimuli and regulate processes such as spore development, competence, and biofilm formation [29], [30], [31]. XCV1925 and XCV1926 overlap by 4 bp, while XCV1926 and *acnB* are separated by a 50-bp intergenic region.

**Figure 2:**
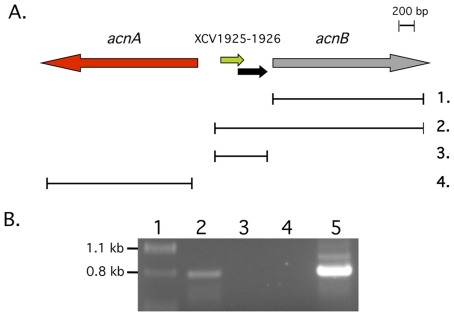
Co-transcription of *xcv1925, xcv1926* and *acnB*. A. Schematic representation of the deletions introduced in the genes at the *acnB* locus. 1. represents the extent of the deletion in strain 85-10Δ*acnB*; 2. represents the deletion in 85-10ΔXCV1925-26*acnB*; 3. represents the deletion in strain 85-10ΔXCV1925-26; and 4. represents the deletion in strain 85-10Δ*acnA*. B. RT-PCR analysis of the XCV1925-XCV1926*-acnB* transcript. Total RNA was isolated and analyzed as described in the Methods using oligonucleotide primers r-secacnB and f-sec3565 (Table S1). Lane 1, DNA size standards; lane 2, PCR product with cDNA; lane 3, control in which RT was omitted from the cDNA synthesis reaction; lane 4, control in which total RNA was omitted from the cDNA synthesis reaction; lane 5, PCR using genomic DNA as template.

To determine whether the XCV1925-XCV1926*-acnB* genes are co-transcribed and form an operon we performed RT-PCR (reverse transcriptase-polymerase chain reaction) with total RNA isolated from *Xcv* strain 85-10 grown aerobically in shake-flask culture to the mid-exponential phase of growth in complex NYG (nutrient yeast glycerol) medium. A 790-bp cDNA fragment spanning *acnB*, XCV1925 and XCV1926 was amplified, suggesting that all three genes are co-transcribed (Fig. 2A). This proposal is furthermore supported by the findings of a recent global transcriptome analysis of *Xcv* [32] in which the authors identified a single cDNA species encompassing the XCV1925, XCV1926 and *acnB* genes. Taken together, these findings suggest that a putative functional relationship exists between XCV1925, XCV1926 and AcnB and therefore we decided to direct the main focus of our study on the AcnB enzyme.

### Expression of *AcnA* and *AcnB* Occurs in Both Exponential and Stationary Phase Cultures

**Figure 3:**
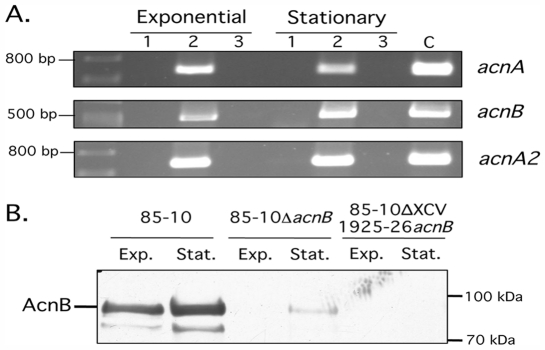
Analysis of aconitase transcripts and AcnB protein levels at different stages of growth. A. Semi-quantitative RT-PCR analysis of *acnA, acnB* and *acnA2* transcripts was performed using total RNA isolated from exponential and stationary phase cultures of strain 85-10 grown *in vitro* in MA minimal medium. Equivalent amounts of RNA were used for cDNA synthesis using oligonucleotide primers r-acnB-RT1, r-acnA-RT1 and r-acnA2-RT1 for *acnB*, *acnA* and *acnA2* transcripts, respectively. PCR (33 cycles) was subsequently performed with the respective primer pairs of f-acnB-RT/r-acnB-RT2, f-acnA-RT/r-acnA-RT2 and f-acnA2-RT/r-acnA2-RT2. The lanes represents minus cDNA (lane 1), including cDNA (lane 2) and minus reverse transcriptase (lane 3). The lane labelled C represents the products of a PCR with genomic DNA as template and the primer pairs described above. The lane on the left of each gel segment shows a DNA size standards. Analysis of 16S rRNA revealed equvalent loading (data not shown). B. A Western blot is shown in which 25 µg of protein derived from crude extracts of the strains indicated were separated in 8% SDS-PAGE and transferred to nitrocellulose membranes and subsequently probed with antibodies raised against *E. coli* AcnB. The location of AcnB is indicated and Exp. and Stat. represent samples from exponential and stationary phase cultures, respectively. The location of molecular mass markers in kDa is indicated on the right of the Figure.

In order to analyze when the Acns of *Xcv* are expressed during *in vitro* growth, a semi-quantitative RT-PCR analysis of the *acnB* transcript, and as a control the *acnA* transcript, was performed. Total RNA was isolated from exponential and stationary phase cultures of *Xcv* growing in rich medium and aliquots were analyzed by RT-PCR (Fig. 3A). The results show that transcripts from both *acnB* and the divergently transcribed *acnA* genes were detectable in both stages of growth. Slightly reduced levels of the *acnA* transcripts were detected in stationary phase cultures compared with exponentially growing cells, while the opposite was the case for *acnB* transcript levels. As a further control we analyzed the transcript levels of a second *acnA* gene (XCV1158), termed *acnA2*, which is located elsewhere on the chromosome of *Xcv*, and which encodes a predicted methylcitrate dehydratase that showed similar levels of transcript in both exponential and stationary phase cells and thus acted as a loading control (Fig. 3A).

In addition to transcript studies, we analyzed the amounts of AcnB protein by immunoblotting in *Xcv* cells from exponential or stationary phase cultures. As the putative AcnB protein from *Xcv* shares 72% overall amino acid identity and 84% similarity with the deduced amino acid sequence of AcnB from *E. coli*, we wondered whether both proteins might share immunogenic epitopes. Indeed, we could show that anti-AcnB antibodies from *E. coli* cross-reacted with an approximately 92-kDa polypeptide in extracts of *Xcv* (Fig. 3B), which is in close agreement with deduced molecular weight of AcnB from *Xcv* of 92,659. The amount of AcnB in extracts derived from stationary phase cultures was similar to that in extracts from exponential phase cells. A second cross-reacting polypeptide that migrated with an approximate molecular mass of 86-kDa was also detected. This polypeptide possibly represents a degradation product of full-length AcnB (Fig. 3B).

In order to demonstrate that the cross-reacting polypeptide was indeed AcnB from *Xcv* we constructed two distinct *acnB* deletion mutants of *Xcv* (Fig. 2A), Strain 85-10Δ*acnB* has a deletion encompassing codon 1 to the termination codon of the *acnB* gene, while strain 85-10ΔXCV1925-26*acnB* carries a deletion in the complete XCV1925, XCV1926 and *acnB* genes (see Methods; Table 1). The strong cross-reacting 92-kDa polypeptide was absent in extracts derived from strains 85-10Δ*acnB* and 85-10ΔXCV1925-26*acnB*, thus demonstrating that this polypeptide indeed represented AcnB from *Xcv* (Fig. 3B). The weak, cross-reacting polypeptide observed in extracts from stationary phase cells of strain 85-10Δ*acnB* that migrated at a size similar to AcnB has yet to be identified. Notably, this cross-reacting polypeptide was not observed in extracts derived from strain 85-10ΔXCV1925-26*acnB* (Fig. 3B), suggesting that XCV1925 or XCV1926 might directly or indirectly affect its synthesis. Taken together, these results demonstrate that AcnB is present in *Xcv* cells throughout the *in vitro* growth phase.

**Table 1:** Strains and plasmids used in this study.

Strains/plasmids	Genotype or relevant characteristic	Reference/Source
*Xcv* strains		
85-10	Pepper-race 2; wild type; Rif^R^	[52], [58]
85*	85-10 derivative containing the hrpG* mutation	[40]
85*Δ*hrcN*	85* derivative, deletion of codon 13-432 of *hrcN*	[33]
85-10Δ*acnB*	85-10 derivative, deletion of the complete *acnB* gene	This study
85-10ΔXCV1925*-*26*acnB*	85-10 derivative, deletion of XCV1925, XCV1926, *acnB*	This study
85-10ΔXCV1925-26	85-10 derivative, deletion of XCV1925, XCV1926	This study
85-10Δ*acnA*	85-10 derivative, deletion of the complete *acnA* gene	This study
85-10Δ*hrpG*	85-10 derivative, deletion of *hrpG*	[40]
85-10*Δ*hrpX*	85* derivative, deletion in *hrpX*	[38]
*E. coli* strains		
W3110	Prototroph	Laboratory collection
JRG3258	W3110 *acnB*::tet^R^	[45]
DH5αpir	F^-^ *recA hsdR17*(r_k_^–^,m_k_^+^) *Φ80dlacZ ΔM15*	[59]
XL1 blue	Tet^R^ *recA*1 *endA*1 *gyrA*96 *thi-1 hsdR*17 *supE*4 *relA*1 *lac*[F^-^ *proAB lacI^q^Z*ΔM15 Tn*10*]	Stratagene
Plasmids		
pBlueskript(II)KS	Phagemid, pUC derivate, Ap^R^	Stratagene
pOK1	Suicide vector *sacB sacQ mobRK2 oriR6K* Sm^R^	[54]
pLAFR6	RK2 replicon, Mob^+^ Tra^–^, multicloning site flanked by transcription terminators, Tc^R^	[60]
pL6*acnB*	pLAFR6 carrying *acnB* from *Xcv*	This study
pBRM	cloning vector, *bla* (Ap^R^), Golden gate-compatible derivate of pBBR1MCS-5, *lacPOZ*’	[46]
pBRM*acnB*	pBRM carrying *acnB* from *Xcv*	This study

### Aconitase B is not Required for *in Vitro* Growth of *Xcv* when Sucrose is the Carbon Source

**Figure 4:**
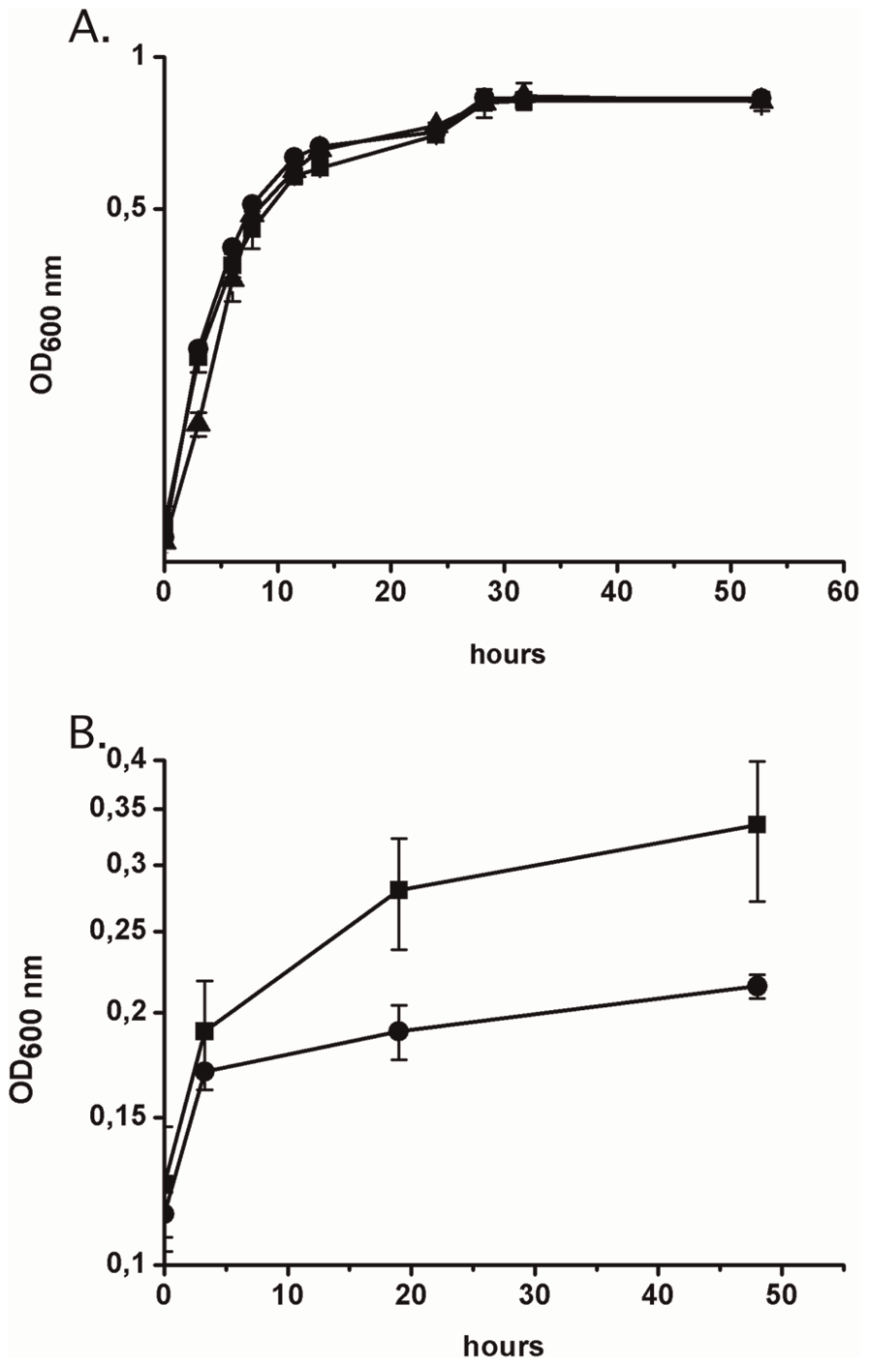
Strain 85-10Δ*acnB* shows restricted growth *in vitro* with citrate as a carbon source. The indicated strains were grown as follows: A. Aerobic growth *in vitro* in MA minimal medium with sucrose as carbon source, where filled circles represent strain 85-10, open circles represent strain 85-10Δ*acnB* and filled inverted triangles represent strain 85-10ΔXCV1925-26*acnB*; B. Growth of strain 85-10 (filled squares) and 85-10Δ*acnB* (filled circles) aerobically in MA minimal medium with 15 mM citrate as sole carbon source. The standard error is shown for each experiment.

Two further mutant derivatives of *Xcv* strain 85-10 were constructed in which the *acnA* gene or the two small genes XCV1925 and XCV1926 were deleted (see Fig. 2 and Methods). The resulting mutant strains 85-10Δ*acnA* and 85-10ΔXCV1925-26, along with strains 85-10 (wild-type), 85-10Δ*acnB* and 85-10ΔXCV1925-26*acnB*, all grew with similar rates and attained similar final optical densities *in vitro* in NYG medium (data not shown). Growth studies performed in minimal medium with sucrose showed that the strains 85-10, 85-10Δ*acnB* and 85-10ΔXCV1925-26*acnB* also showed similar growth phenotypes (Fig. 4A). Moreover, strain 85-10Δ*acnA* also grew like the wild-type under these conditions (data not shown). The lack of an *in vitro* growth phenotype for the 85-10Δ*acnA* mutant is in agreement with previous observations for an *rpfA* (*acnA*) mutant of *X. campestris* pv. *campestris* [23].

### *Xcv* Strain 85-10Δ*acnB* Shows Restricted Growth in Minimal Medium with Citrate

As citrate is the substrate for aconitase and the bacterium encodes a citrate transporter, we next compared growth of strains 85-10 and 85-10Δ*acnB in vitro* in minimal medium with 15 mM citrate as sole carbon source (Fig. 4B). The wild-type strain 85-10 grew more poorly than with sucrose as a carbon source (compare Fig. 4A and Fig. 4B) but it nevertheless attained a final optical density at 600 nm of approximately 0.35. Without addition of a carbon source no growth of the wild-type was observed (data not shown). Strain 85-10Δ*acnB* showed a clearly reduced ability to grow with citrate compared with the wild-type strain (Fig. 4B). Taken together, the findings of the *in vitro* growth studies indicate that, although the *acnB* mutation did not affect growth with sucrose as a carbon source growth in the presence of citrate was affected.

### Strains Lacking *AcnB* Show Delayed Growth and Symptom Formation in Pepper Plants

**Figure 5:**
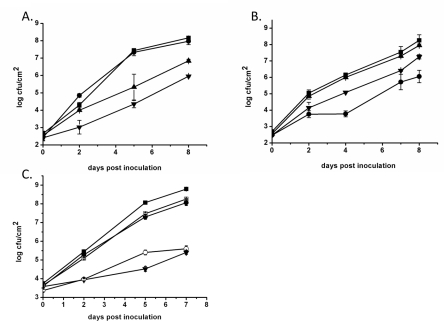
Strain 85-10Δ*acnB* shows restricted growth *in planta*. The indicated strains were grown as follows: A. Growth *in planta* where filled squares represent strain 85-10, filled circles represent strain 85-10Δ*acnA*, filled triangles represent strain 85-10Δ*acnB* and filled inverted triangles represent strain 85*Δ*hrcN* B. Growth *in planta* where filled squares represent strain 85-10, filled triangles represent strain 85-10ΔXCV1925-26, filled inverted triangles represent strain 85-10ΔXCV1925-26*acnB* and filled circles represent strain 85*Δ*hrcN*; C. Growth *in planta* where filled squares represent strain 85-10, open squares represent strain 85-10/pLAFR6, filled circles represent strain 85-10Δ*acnB*/pL6*acnB*, open circles represent strain 85-10Δ*acnB*/pLAFR6 and filled inverted triangles represent 85*Δ*hrcN*. The standard error is shown for each experiment.

Citrate is abundant in the tomato apoplast [8] and recent studies have shown that expression of the *citH* gene of *Xcv*, encoding a citrate transporter, is up-regulated *in planta* [9], [10]. Therefore, we investigated the consequences of the different gene deletions on growth of the respective bacterial strain *in planta*. Strains 85-10, 85-10Δ*acnA*, 85-10Δ*acnB*, 85-10ΔXCV1925-26 and 85-10ΔXCV1925-26*acnB* were inoculated into leaves of the susceptible pepper line Early California Wonder (ECW). Strain 85-10Δ*acnA* and the wild type 85-10 showed similar growth in susceptible plants (Fig. 5A). As a negative control we analyzed growth of strain 85-10Δ*hrcN* [33], which lacks the ATPase HrcN of the T3S system and was therefore strongly impaired in *in planta* proliferation. In contrast to the growth phenotypes of strains 85-10 and 85-10Δ*acnA*, strain 85-10Δ*acnB* displayed a clearly reduced ability to grow in the plant apoplast (Fig. 5A). The growth of the *acnB* mutant was, however, not as strongly reduced compared with growth of the *hrcN* mutant. In a similar experiment, growth of strains 85-10ΔXCV1925-26 and 85-10ΔXCV1925-26*acnB* were compared with growth of strain 85-10 (Fig. 5B). Strain 85-10ΔXCV1925-XCV1926-*acnB* showed a similarly reduced growth phenotype to that observed for strain 85-10Δ*acnB*. In contrast, however, strain 85-10ΔXCV1925-26 grew like the wild-type strain 85-10 (Fig. 5B). This result indicated that the reduced growth phenotype of strain 85-10ΔXCV1925-26*acnB in planta* was caused solely by the *acnB* mutation and that the additional deletion of the XCV1925-XCV1926 genes had no effect on growth.

The reduced growth of strain 85-10Δ*acnB in planta* could be complemented by introduction of plasmid pL6*acnB*, which encodes AcnB into the mutant (Fig. 5C). The slightly impaired growth *in planta* we observed for strains containing the plasmid pLAF6 accounted for the similarly poor growth phenotype of 85-10Δ*acnB*/pLAFR6 and 85*Δ*hrcN*. Taken together these results indicate that AcnB is required for optimal growth of *Xcv* in susceptible pepper plants.

### Strains Lacking *acnB* Show Delayed Appearance of Disease Symptoms

Strains 85-10Δ*acnB* and 85-10ΔXCV1925-26*acnB* were next analyzed to determine whether they were affected in the induction of disease symptoms in the ECW pepper line or in the ability to induce the HR (hypersensitive response) in the resistant pepper line ECW-10R [34], [35]. ECW-10R pepper plants carry the *Bs1* resistance gene and recognize the effector protein AvrBs1, which is delivered by the T3SS of strain 85-10 [34], [36]. The HR is a rapid local cell death at the infection site that restricts bacterial ingress and is activated upon detection of individual effector proteins (also designated Avr [avirulence] proteins) by the plant surveillance system [37].

**Figure 6:**
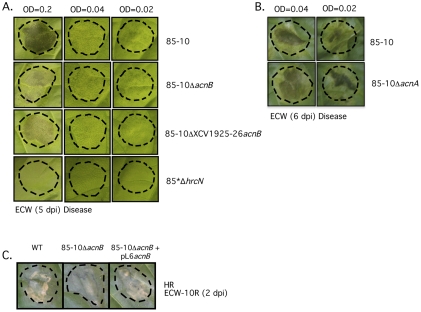
Mutants lacking aconitase B exhibit delayed appearance of disease symptoms and the HR. Disease symptoms (A, B) and the hypersensitive response (HR) (C) induced by *Xcv* wild-type and *acnB* mutant strains were analyzed. In panel A strains 85-10, 85-10Δ*acnB*, 85-10ΔXCV1925-26*acnB*, and 85*Δ*hrcN* were infiltrated into leaves of susceptible Early Cal Wonder (ECW) at the different initial optical densities indicated (OD_600nm_ of 0.2  =  2 × 10^7^ CFU ml^–1^), while in panel B symptoms generated by growth of strains 85-10 and 85-10Δ*acnA* were compared. Disease symptoms were photographed five (A) or six (B) days after infiltration. Dashed lines indicate the infiltrated areas. Strain 85*Δ*hrcN* lacks a functional T3SS, shows no disease symptoms and acted as a negative control. In panel C strains 85-10Δ*acnB* and 85-10Δ*acnB*(pL6*acnB*) were infiltrated into leaves of resistant ECW-10R pepper plants using an OD_600nm_ of 0.1 (1 × 10^7^ CFU ml^−1^). Plasmid pL6*acnB* encodes AcnB. HR was documented 2 days after infiltration.

Strains 85-10Δ*acnB* and 85-10ΔXCV1925-26*acnB* displayed a slightly reduced and delayed appearance of disease symptoms in susceptible plants (Fig. 6A), which was most apparent when the initial inoculum used for infiltration was low (2 × 10^7^ colony forming units ml^–1^;OD = 0.2) (Fig. 6A). This phenotype was only subtle and probably reflected the slower growth of the mutant strain *in planta* compared with the wild-type. Strain 85-10Δ*acnA* had the same disease symptom phenotype as the wild-type strain 85-10 (Fig. 6B), consistent with both strains showing similar growth *in planta* (see Fig. 5A).

Infiltration of ECW-10R pepper plants with strain 85-10Δ*acnB* also resulted in a slight reduction in HR in resistant plants when compared with the wild-type strain 85-10 (Fig. 6C). The phenotype of 85-10Δ*acnB* could be complemented by introduction of plasmid pL6*acnB* (Fig. 6C).

Strain 85-10ΔXCV1925-26 had phenotypes comparable with those of wild type 85-10 with regard to disease symptoms and the HR (data not shown).

### The Level of AcnB is Unaffected by the *hrp* Regulators HrpG and HrpX

**Figure 7:**
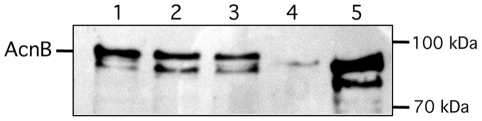
Aconitase B synthesis is unaffected by the T3SS regulators HrpG and HrpX. Western blot analysis of crude extracts (25 µg of protein in each) derived from the strains indicated separated on 8% SDS-PAGE and transferred to nitrocellulose. Aconitase B was detected by antibodies raised against *E. coli* AcnB. Lane 1, 85-10; lane 2, 85-10Δ*hrpG*; lane 3 85*Δ*hrpX*; 85-10Δ*acnB*; *E. coli* W3110 (5 µg of protein from a crude extract). Because no change in protein levels was observed no loading control was shown. Molecular mass markers (Pageruler Prestained Protein Ladder, Thermo Scientific) are given in kDa.

It was previously shown that the OmpR-type regulator HrpG (HR and pathogenicity) regulates the expression of a genome-wide regulon including T3SS and putative virulence genes [1], [38], [39], [40]. Strain 85* contains a constitutively active derivative of HrpG, HrpG*, which leads to the constitutive expression of T3SS genes [38], [40]. As well as being regulated by HrpG, synthesis of the T3SS is also regulated by *hrpX* [1]. The *hrpX* gene encodes an AraC-type transcriptional regulator [41], [42]. In order to test whether expression of the XCV1925-XCV1926*-acnB* operon was influenced by the HrpX or HrpG regulators, the levels of the AcnB polypeptide were analysed in *hrpX* and *hrpG* deletion mutants. Western blot analysis revealed that AcnB levels were unaltered in either mutant strain compared with strain 85-10 (Fig. 7).

### Strains Lacking Aconitase B are more Sensitive Towards the Superoxide-generating Compound Menadione

**Figure 8:**
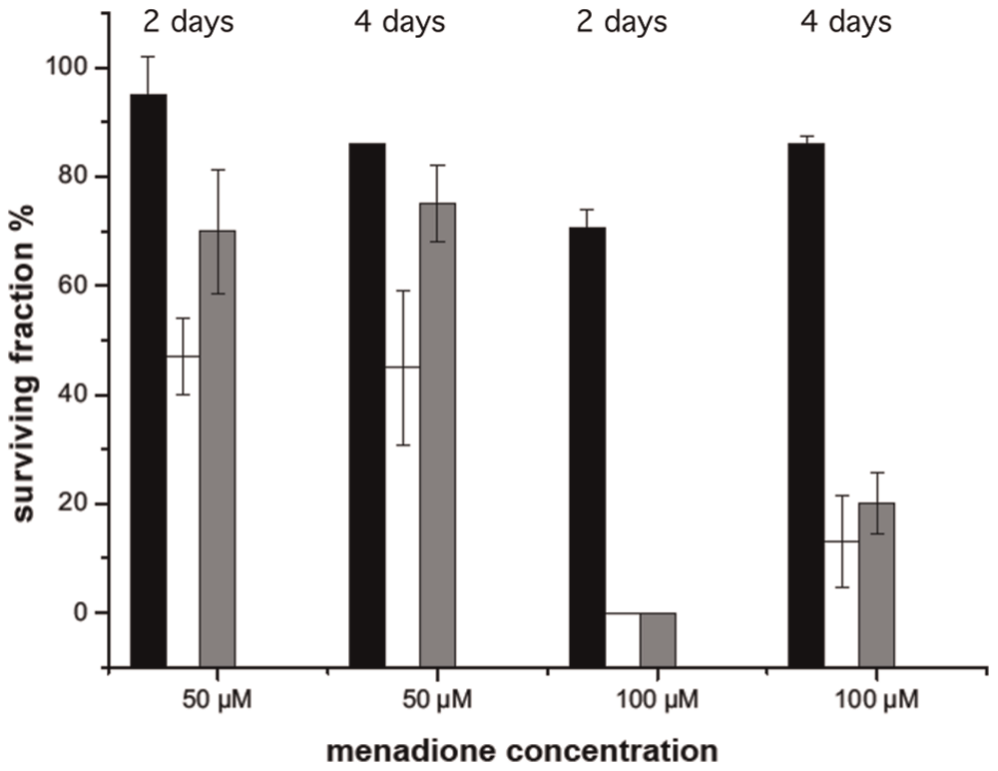
*X. campestris* pv. *vesicatoria* strain 85-10Δ*acnB* has increased sensitivity to the superoxide-generating agent menadione. Dilutions of 10^−6^ of exponential phase cultures (OD_600_ =  0.6) of the *Xanthomonas* strains indicated were spotted on NYG agar plates containing 50 µM and 100 µM menadione. Bacterial colonies surviving the treatment were counted after 24 h and 48 h of incubation at 30°C and CFU were expressed as surviving fraction in percent. Strain 85-10 (black columns), strain 85-10Δ*acnB* (white columns) and strain 85-10ΔXCV1925*-26acnB* (gray columns).

Aconitase B enzymes have a [4Fe-4S] cluster that is sensitive to oxidative stress [12], [13], [43]. Exposure of strains 85-10, 85-10Δ*acnB* and 85-10ΔXCV1925-26*acnB* to the superoxide-generating chemical menadione revealed that both *acnB* mutants were more sensitive to 50 µM menadione than the wild-type strain 85-10 (Fig. 8). Increasing the concentration of menadione to 100 µM made this difference much more apparent and while for the wild-type strain 85-10 after 4 days^’^ incubation 90% of the bacteria survived, survival of both mutants was severely impaired and attained levels of between 10 and 20% (Fig. 8). This result suggests that AcnB might have a role in either sensing changes in superoxide levels or protecting *Xcv* from the deleterious effects of oxidative stress. Notably, *E. coli acnB* mutants also show increased sensitivity toward superoxide-generating chemicals such as methyl viologen [44]. In contrast to the phenotype of an *E. coli acnB* mutant, however, strain 85-10Δ*acnB* failed to reveal a difference in survival when compared with strain 85-10 after exposure to 5 mM hydrogen peroxide (data not shown). These findings demonstrate that although certain phenotypes of *Xcv acnB* mutants are common to those reported for other bacteria, nevertheless, clearly different phenotypes are evident for the *Xcv acnB* mutants.

### AcnB from *X. Campestris* pv. *Vesicatoria* is functional in *E. coli*

**Figure 9:**
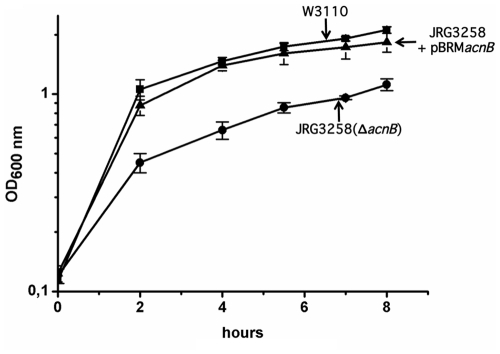
Aconitase B from *Xcv* functionally complements an *E. coli acnB* mutation. The *E. coli* wild type W3110 (filled squares), the *acnB* mutant JRG3258 (filled circles) and JRG3258 transformed with plasmid pBRM*acnB* (filled triangles) were grown aerobically in LB broth as described in the Methods section. The standard error is shown for each experiment.

In many bacteria aconitases are differentially regulated in response to the prevailing growth conditions. For example, in *E. coli* AcnB is the main TCA cycle enzyme and it is functional in the exponential phase of growth, while AcnA is switched on during oxidative stress and in the stationary phase [21], [44]. Because of the strong dependence on AcnB during the exponential growth phase *E. coli acnB* mutants show a reduced growth phenotype in liquid culture, while *acnA* mutants show no growth phenotype [45]. This phenotype provided the opportunity to demonstrate whether AcnB from *E. coli* and *Xcv* are functionally interchangeable. For this, the *acnB* gene from *Xcv* was cloned under control of the *lac* promoter in-frame with a C-terminal c-Myc epitope-encoding sequence into the expression vector pBRM [46] (see Methods). The resulting plasmid pBRM*acnB*, when introduced into an *E. coli acnB* mutant, restored the aerobic growth rate of the mutant in liquid culture to a rate similar to that of the wild type (Fig. 9). This result indicates that the two AcnB enzymes are functionally interchangeable and that the C-terminal c-Myc-tag on AcnB_Xcv_ did not impair enzyme function. The lack of an observable *in vitro* growth phenotype for strain 85-10Δ*acnB*, however, contrasts sharply the growth phenotype of an *E. coli acnB* mutant [45] (see also Fig. 3).

## Discussion

The findings of this study demonstrate that the AcnB enzyme of *Xcv* is required for optimal growth of the bacterium in the apoplast of pepper plants. This is of significance because *acnB* mutants of *Xcv* show no growth phenotype when they are grown *in vitro* in shake cultures in the presence of sugar substrates. Reduced growth in pepper plants suggests that the bacterium utilizes citrate as one of its carbon sources *in planta*. Although not analyzed so far for *Xcv*, apoplast metabolite studies performed with tomato have identified citrate, along with succinate, as the main organic acid present in apoplastic fluid [8]. Both of these substrates require the TCA cycle to enter primary metabolism. Moreover, the importance of citrate as an apoplastic substrate of *Xcv* is exemplified by the findings of two studies. In the first, expression of the *citH* gene, encoding a citrate transporter in *Xcv*, was shown to be up-regulated specifically in tomato [9]. In a more recent second study CitH was demonstrated to be required for citrate uptake by the bacterium when it is growing in tomato [10]. Taken together, these observations are consistent with citrate, the substrate of the AcnB enzyme, being a carbon source for *Xcv* in apoplast fluid. Our demonstration in this study that *in vitro* growth of the *acnB* mutant with citrate was less efficient than the wild type supports these findings. The availability of TCA cycle intermediates as carbon sources *in planta* would also explain why plant-pathogenic *Xanthomonas* species are obligate aerobes, because anaerobic bacteria cannot metabolize these compounds effectively.

The delayed appearance of disease symptoms in the *Xcv acnB* mutant is a subtle phenotype that is possibly related to the reduced growth of the mutant in the plant apoplast. However, a reduced growth rate does not explain the reduction in the HR phenotype of the *acnB* mutant. This may be linked to more general effects on the bacterium’s physiology caused by the impaired citric acid cycle of the *acnB* mutant. The *Xcv acnA* mutant failed to exhibit either a growth or virulence phenotype *in planta*, which highlights a potential difference between *Xcv* and the *Xcc* pathovar [23].

A further potentially important function for aconitases in pathogenic bacteria is as sensors of oxidative stress, conditions which are often prevalent during host-microbe interactions [6], [47], [48]. Sensitivity of these enzymes towards oxidative stress is mediated through the iron-sulphur cluster in aconitase, which has an essential catalytic function in allowing citrate conversion to isocitrate. Our observation here that *acnB* mutants of *Xcv* are more susceptible toward superoxide-generating chemicals would be consistent with a role for this enzyme in sensing the presence of ROS *in planta* and possibly in maintaining iron homeostasis [6].

Finally, although we failed to demonstrate under the conditions tested in this study that the aconitase A of *Xcv* is essential for optimal growth *in planta*, as has been suggested in a previous study with *X. campestris* pv. *campestris* [23], our findings nevertheless show that in the absence of AcnB a further aconitase is likely to be functional *in vitro* because an *acnB* mutant retained the abililty to grow slowly with citrate while the wild-type failed to grow without carbon source supplementation. Whether this is AcnA or AcnA2 will require the analysis of double knock-out mutants that lack AcnB and either one of the predicted AcnAs.

In summary our findings underline the general importance of aconitases in plant-microbe interaction. The importance of aconitase B to *Xcv* might reflect multiple functions of the protein *in planta*: 1. Aconitase B is a key enzyme for the metabolism of TCA intermediates; 2. AcnB possibly has a role in sensing oxidative stress; 3. Although not tested in this study, it is conceivable that aconitase B is involved in monitoring iron homeostasis during growth of the bacterium in the plant. Future studies will focus on elucidating whether AcnB indeed performs all of these functions when the bacterium is growing in pepper plants.

## Methods

### Bacterial Strains, Growth Conditions, and Plasmids

The bacterial strains and plasmids used in this study are described in Table 1. *X. campestris* pv. *vesicatoria* strains were cultivated at 30°C in complex nutrient-yeast-glycerol (NYG) medium [49] or in minimal medium A [50] supplemented with sucrose (10 mM) and casamino acids (0.3% w/v). *E. coli* cells were cultivated at 37°C in Luria-Bertani medium. Plasmids were introduced into *E. coli* by electroporation or using heat-shock treatment and then into *Xcv* by conjugation using pRK2013 as a helper plasmid in triparental matings [51]. Antibiotics were added to culture media at the following final concentrations: ampicillin 100 µg/ml, kanamycin 25 µg/ml, rifampicin 100µg/ml, spectinomycin 100µg/ml, tetracycline 5µg/ml, cyclohexamide 50µg/ml.

### Plant Material and Plant Inoculations

The near-isogenic pepper cultivars Early Cal Wonder (ECW) and ECW-10R [52], which contains the *Bs1* resistance gene [35], were grown and inoculated with *Xcv* as described [53]. Bacteria were inoculated into the intercellular spaces of fully expanded leaves of 5–6 week-old plants using a needleless syringe at a concentration of 10^7^ CFU ml^–1^ (OD  =  0.01) to 4 × 10^8^ CFU ml^–1^ (OD  =  0.4) in 10 mM MgCl_2_ unless stated otherwise. The appearance of disease symptoms was scored over a period of 5 days after inoculation and the HR over a period of 2 days.

For *in planta* growth curves, bacteria were inoculated at a density of 10^4^ CFU/ml into leaves of susceptible pepper ECW plants (age of 5–6 weeks). Bacterial growth was examined as described [53]. Experiments were repeated at least three times and each time with three separate plants.

### Construction of Deletion Mutants

To delete genes, approximately 1.0 kb of DNA sequences flanking the gene of interest were amplified by PCR using the primers listed in Table S1 and with *Xcv* genomic DNA as template. The PCR products to delete *acnB* were digested with XbaI/HindIII and Hind III/ApaI and the products to delete XCV1925-XCV1926*-acnB* with XbaI/Hind III and HindIII/SalI, followed by ligation into the XbaI/ApaI and XbaI/SalI sites, respectively, of the suicide plasmid pOK1. Similarly, the XbaI/HindIII- and HindIII/BamHI-digested PCR products to delete XCV1925-XCV1926 and the XbaI/HindIII- and HindIII/ApaI-digested products used to delete *acnA* were ligated into XbaI/BamHI and XbaI/ApaI sites of the pOK1 vector, respectively. The resulting constructs pOKΔ*acnB*, pOKΔ*1925-26acnB,* pOKΔ*1925-26* and pOKΔ*acnA* were conjugated into strain 85-10 as described [54]. Double-crossover events led to generation of strains 85-10Δ*acnB*, 85-10ΔXCV1925-26*acnB,* 85-10ΔXCV1925-26 and 85-10Δ*acnA.* The extent of the *acnB* gene deletion 85-10Δ*acnB* included from 1 bp prior to the translation initiation codon to 3 bp after the terminations codon; the deletion in 85-10ΔXCV1925-26 started 6 bp before the translation initiation codon of XCV1925 and ended 10 bp after the termination codon of XCV1926; the deletion in strain 85-10ΔXCV1925-26*acnB* started 6 bp before the translation initiation codon of XCV1925 and ended 3 bp after the terminations codon of *acnB*; and the deletion in 85-10Δ*acnA* started 2 bp before the translation initiation codon of *acnA* and ended 1 bp after the termination codon.

### Construction of Plasmids

To enable complemention studies, the *acnB* and XCV1925-XCV1926*-acnB* genes were amplified by PCR using the primers listed in Table S1 and genomic DNA from *Xcv* as template. The PCR products were digested with XbaI/BamHI (in the case of *acnB*) and HindIII/XbaI (in the case of XCV1925-XCV1926*-acnB*) and ligated into the XbaI/BamHI and HindIII/XbaI sites, respectively, of pLAFR6 vector. The resulting constructs were transformed in strain *E. coli* XL1 blue. These transformed *E. coli* strains were used as donors to conjugate pL6*acnB* into strain 85-10Δ*acnB* and 85-10ΔXCV1925-26-*acnB.* The *acnB* gene was also cloned into the Gloden-Gate-compatible vector pBRM by amplification with primers f-pBRM-acnB and r-pBRM-acnB listed in Table S1 and incorporation of appropriate BsaI restriction sites to introduce a C-terminal c-Myc epitope onto AcnB [46].

### Immunoblot Analyses

For detection of AcnA and AcnB in crude extracts of *Xcv*, bacteria were cultivated in NYG or minimal media as described above. Samples of cells were harvested from mid-exponential or stationary phase cultures. After cell harvest, cells were resuspended in 2–3 ml of MOPS buffer pH 7.0 and lysed on ice by sonication (30W power for 5 minutes with 0.5 sec pulses). Unbroken cells and cell debris were removed by centrifugation for 15 min at 10 000 × g at 4°C and the supernatant was used as the crude cell extract. Protein concentration was determined as described [55] and aliquots of 50 µg of protein from the crude extract were separated by SDS-polyacrylamide gel electrophoresis (PAGE) using 10% (w/v) polyacrylamide [56] and transferred to nitrocellulose membranes as described [57]. Aconitase was identified using polyclonal antiserum raised against AcnB from *E.coli* (a kind gift from J. Green, Sheffield, UK) or monoclonal anti-c-Myc antibodies (Roche Applied Science, Mannheim, Germany). Anti-AcnB antiserum was used at a dilution of 1∶10,000. Horseradish-peroxidase-labeled conjugate (Goat Anti-Rabbit IgG (H+L)–HRP-conjugate, 1∶5000; Biorad, Munich, Germany) was used as secondary antibody-conjugate and the reaction was visualized by enhanced chemiluminescence (Roche Diagnostics, Mannheim, Germany). Immunoblots were performed at least twice, each time with freshly prepared samples.

### RT-PCR Analysis

Isolation of total RNA for reverse transcriptase PCR (RT-PCR) analysis after growth of the bacteria in NYG medium. RNA extraction and cDNA synthesis were performed as described previously [38]. The transcripts were amplified by PCR using the gene-specific primers listed in Table S1. Experiments were performed minimally two time and each time with freshly isolated RNA samples.

### Complementation of an *E. coli acnB* Mutant with *acnB* from*Xcv*

Analysis of the ability of *acnB* from *Xcv* to complement an *E. coli acnB* mutation we transformed strain JRG3258 (W3110::*acnB*) [45] with pBRM*acnB*. The growth of all strains were analysed in LB medium over 8 h. Experiments were repeated at least three times and each time in triplicate.

### Plate-sensitivity Assay

The resistance level of the cells to menadione was determined using a modification of the plate-sensitivity assay described previously [47]. Dilutions of 10^−6^ and 10^−7^ of exponential phase cultures (OD_600_ =  0.6) of the *Xanthomonas* strains to be tested were spotted on NYG agar plates containing 50 µM or 100 µM menadione (Sigma, Munich). Bacterial colonies surviving the treatment were counted after 24 h and 48 h of incubation at 30°C and data are expressed as surviving fraction in percent. The percentage survival was calculated by dividing the number of CFU from plates with menadione by the number of CFU from control plates without menadione. This experiment was performed in triplicate and repeated at 3 times.

## Supporting Information

Table S1 **Oligonucleotide primers used in this study.**
(DOC)
