# Supplementary material for: Aconitase B Is Required for Optimal Growth of Xanthomonas campestris pv. vesicatoria in Pepper Plants
Source: PLoS One. 2012 Apr 6;7(4):e34941. doi: 10.1371/journal.pone.0034941 (PMC3321045; doi:10.1371/journal.pone.0034941)
Supplement: Table S1 — Oligonucleotide primers used in this study. (DOC) [file pone.0034941.s001.doc]

**Table S1.**

| Primer | Nucleotide sequence | Comment |
| --- | --- | --- |
| f-acn2823Xba | 5- cct agt tct aga ggc gta gca ggt tct cca gca-3' | *acnB* deletion |
| r-xcv1926-3800Hind | 5'-ctgg gta aag ctt cgg gtt ctc caa aaa agc agc-3' | *acnB* deletion |
| f-xcv1928-2825Hind | 5'- ctc act aag ctt tcc cgc cgc tct acc act gcc-3' | *acnB* deletion |
| r-xcv1928-3761Apa | 5' -taa ctg ggg ccc agc ccg acct cct gtc cgt aaa-3’ | *acnB* deletion |
| f-acnA2118Xba | 5'- tgc ata tct aga cga cga act tgc cca ccaccc-3' | *xcv1925-26acnB* mutant |
| r-acn3089Hind | 5'- ctc tga aag ctt tgc tcc aga aag tat gca cgc-3' | *xcv1925-26acnB* mutant |
| r-xcv1928-3761Sal | 5'- taa ctg gtc gac agc ccg cct cct gtc cgt aaa- 3' | *xcv1925-26acnB* mutant |
| f-xcv1928-2825Hind | 5'- ctc act aag ctt tcc cgc cgc tct acc act gcc-3’ | *xcv1925-26acnB* mutant |
| f-acnA2118Xba | 5'-tgc ata tct aga cga cga act tgc cca cca ccc-3' | *xcv1925-26* mutant |
| r-acn3089Hind | 5'-ctc tga aag ctt tgc tcc aga aag tat gca gcg-3' | *xcv1925-26* mutant |
| f-acn3781Hind | 5'-ctg tac aag ctt cgc ttt acc acc ttc acc gct-3' | *xcv1925-26* mutant |
| r-acnB4741Bam | 5'- gta cca gga tcc aca tcg ccg tgc tcc atc tgc-3' | *xcv1925-26* mutant |
| r-Del-acnA-Apa | 5'- aga tgt ggg ccc cgt cgt cga cgc cgg ccg gca-3' | *acnA* mutant |
| f-Del-acnA-Xba | 5'-cac atg tct aga gtc ttg ccg aag cta tgg tac-3' | *acnA* mutant |
| f-Del-acnA-Hind3 | 5'- tgc ata aag ctt atc act cct atg gca tcg aag-3' | *acnA* mutant |
| r-Del-acnA-Hind2 | 5'- ctg gta aag ctt cac agc gcg cat tgc gct gca-3' | *acnA* mutant |
| f-pLAFRacnB-BamHI | 5'- ttc ctt gga tcc ccc tga tcc acg ttc gcg ctt-3' | Cloning of *acnB* in pLAFR6 |
| r-acnB-HindIII | 5'- ccc aag ctt ccc aaa ccc gca ccc tga tcc-3' | Cloning of *acnB* in pLAFR6 |
| f-pBRM-acnB | 5'-ttt ggt ctc tta tgt tgg aag cct atc gcc acc-3' | Cloning of *acnB* in pBRM |
| r-pBRM-acnB | 5'-ttt ggt ctc tca ccg gcg gcg acg gtc tct gcc-3' | Cloning of *acnB* in pBRM |
| r-acnB-RT1 | 5'- cga cgg tct ctg cca cgt cct gat att cct gga -3' | *acnB* cDNA synthesis |
| r-secacnB | 5’- agg tcg tcg gta ttg gtt tcg – 3’ | *acnB* cDNA synthesis |
| r-acnA-RT1 | 5' - tgc gtg ccg cca gtt gcc gca gca cat act gca -3' | *acnA* cDNA synthesis |
| r-acnA2-RT1 | 5' -tgc ctc aag aaa gtc ctg cgc aaa ccg ctg caa -3' | *acnA2* cDNA synthesis |
| f-acnB-RT | 5'- tgc cga ggg cta tgc cga tgc gcg cac cct ggg-3' | RT-PCR of *acnB* |
| r-acnB-RT2 | 5'- tgc cgc cag ttc cgc cga gcc cag gta cac gtt-3' | RT-PCR of *acnB* |
| f-acnA-RT | 5'- gac cat gca ggt ggg caa cgt gga cga cgt gca-3' | RT-PCR of *acnA* |
| r-acnA-RT2 | 5'-gac ggt ggc acg gcg gct ggc gcc gtc ctg cag-3' | RT-PCR of *acnA* |
| f-acnA2-RT | 5'- gtg cgc gca gcg tac gcg gca tgc gcc cgt tgg-3' | RT-PCR of *acnA2* |
| r-acnA2-RT2 | 5'- ccg gca cct gca gtt gct cgc cat tgc gcc ggt-3' | RT-PCR of *acnA2* |
| f-sec3565 | 5'- cca ccc agg aag cgg cgg cag – 3' | RT-PCR of *xcv1925-acnB* |
| f-hrpG-RT | 5'-aac gac cac tcc ccc tcc aat gcc gga tcg gtg-3' | RT-PCR of *hrpG* |
| r-hrpG-RT2 | 5'-tgc gca act tgt aga tgt gct gct cca tgg tgc-3' | RT-PCR of *hrpG* |
